# A Review of Implementation Strategies to Enhance PrEP Delivery for People Experiencing Housing Insecurity: Advancing a Multifaceted High-Touch, Low-Barrier Approach

**DOI:** 10.1007/s11904-024-00714-z

**Published:** 2024-11-22

**Authors:** Jennifer Velloza, Nicky J. Mehtani, Matthew D. Hickey, Elizabeth Imbert, Ayesha Appa, Elise D. Riley

**Affiliations:** 1 Department of Epidemiology & Biostatistics, University of California San Francisco, 550 16th Street 2nd Floor, San Francisco, CA 94158 USA; 2 Department of Medicine, University of California San Francisco, San Francisco, CA USA

**Keywords:** HIV, Pre-exposure prophylaxis, Implementation strategies, Homelessness and unstable housing, Prevention, Care engagement

## Abstract

**Purpose of Review:**

This review summarizes key implementation strategies to advance oral and long-acting PrEP delivery for unstably housed people in the United States.

**Recent Findings:**

People experiencing homelessness and housing instability face barriers to PrEP uptake and adherence including lack of safe medication storage, competing basic needs, insurances issues, and/or mental health or substance use disorders. Recent advancements in HIV treatment and prevention provide evidence on high-touch, low-barrier implementation approaches to address these challenges. We compiled these approaches into a multi-component implementation strategy, “SHELTER”, which includes: low-barrier primary care, case management, incentives, outreach, care coordination, multidisciplinary provider collaboration, data tracking, and robust provider-patient relationships.

**Summary:**

The US has fallen short of our Ending the Epidemic targets, in part due to challenges in PrEP delivery for people experiencing housing instability. SHELTER provides a comprehensive approach for considering critical components of HIV prevention for this population that can be used in future oral and long-acting PrEP programs.

## Introduction

People experiencing homelessness (PEH) and unstable housing face high burdens of HIV acquisition and poor outcomes along the HIV care continuum, which has contributed to shortfalls on Ending the HIV Epidemic goals in the United States [1]. In 2023, 1 of every 500 people in the US were homeless on any given day, and many more faced housing insecurity, including concerns about eviction or property loss [2]. In the US, the prevalence of housing instability has increased by 12% since 2022 and disproportionately affects BIPOC individuals, people with mental health and substance use disorders, and LGBTQ + individuals [2]. The intersectional identities and social and structural systems of oppression among these marginalized groups also drive inequity in HIV risk, morbidity, and mortality in this population [3, 4]. Unstably housed people report a lower likelihood of receiving medical care outside of emergency departments or hospital settings and describe lower levels of knowledge about oral HIV pre-exposure prophylaxis (PrEP) than those who are stably housed [5–11]. Unstably housed people who are also living with HIV have challenges with daily oral antiretroviral therapy (ART) adherence and experience significantly higher rates of viremia and onward HIV transmission than housed people living with HIV [5–11].

Emerging patient-centered models of HIV service delivery have attempted to address these intersecting needs and social determinants of health by providing low-barrier (e.g., no appointment) care with primary care that is augmented by ancillary services (“wrap-around services”). Care models include clinic-based and street-based mobile outreach. To date, these programs have largely focused on ART delivery for people with HIV [12, 13], and gaps persist in providing person-centered, wrap-around models of care for unstably housed people without HIV including PrEP delivery. Many lessons from the HIV treatment space can be built upon and generalized to the PrEP delivery landscape, although unstably housed people without HIV may have particular challenges balancing HIV prevention with other competing demands. While a few low-barrier status-neutral care models for unstably housed people that combine HIV treatment and oral and long-acting PrEP delivery have been described, their outcomes related to improving PrEP knowledge, care linkage, and adherence that reduce HIV acquisition have generally not been reported [14–16].

We are currently in a shifting HIV prevention landscape, with increasing availability of long-acting PrEP modalities and a push toward differentiated service delivery models to broaden PrEP coverage [17]. Ending the HIV epidemic in the United States will require improving uptake of these new prevention approaches to more effectively engage and retain individuals experiencing homelessness and unstable housing. Current person-centered ART and PrEP delivery models include combinations of numerous complex time- and resource-intensive components [18], which have not been well-specified or consistently included and evaluated across models. Key barriers to the widespread utilization and success of differentiated PrEP service delivery for PEH include gaps in: 1) understanding prior and existing care model innovations; 2) identifying a set of core components that may form a multicomponent PrEP implementation approach for this population; and 3) evaluating the effectiveness and cost-effectiveness of components on PrEP and HIV incidence outcomes to ensure that programmatic decisions and priorities align with health outcomes [19]. We conducted a literature review of implementation strategies to promote PrEP for unstably housed people and propose a multi-component PrEP strategy (“SHELTER”) based on the strategies identified in the literature. We also provide exemplary PrEP and ART programs that highlight elements of SHELTER to advance PrEP delivery for this population.

## Theoretical Framing and Review Methods

### Theoretical Framing

A critical first step to identifying core components of a PrEP implementation strategy for unstably housed people is developing a thorough understanding of barriers to oral and long-acting PrEP use in this population and possible approaches to address these barriers. The Behavior Change Wheel (BCW), and associated Capability-Opportunity-Motivation-Behavior (COM-B) model and Theoretical Domains Framework, offers a rigorous implementation science approach to identify behavioral targets of PrEP delivery [20, 21]. We selected this theoretical framing as our organizing approach because it is focused on identifying individual PrEP client, provider, and clinic determinants of behavior. It offers guidance on evidence-based “intervention functions” for each type of determinant that could bring about behavior change and has been widely used for the development of multicomponent interventions and implementation strategies across a variety of health conditions. [12, 20–24]

The BCW is represented as a hub with three central components: capability, opportunity, and motivation [20, 21]. These three broad categories can be further described by constructs from the Theoretical Domains Framework, as shown in the first two columns of Table 1. Based on prior research of determinants of PrEP use among PEH and unstable housing [11, 12, 15, 18, 25–32], we hypothesized several determinants that would need to be addressed to enable PrEP initiation, adherence, and persistence among this population for each COM-B and Theoretical Domains Framework category (column #3 in Table 1). For example, the “physical opportunity” domain of the COM-B framework includes environmental context and access to resources to facilitate PrEP use. Determinants of PrEP initiation, adherence, and persistence around this domain are the ability to reach the clinic, to store PrEP (either in a safe location or with a trusted person), access to a phone to communicate with health care providers, and the ability to have other physical needs met, including food, housing and treatment of mental health and substance use disorders.

**Table 1:** Facilitators and barriers to HIV prevention service engagement among people experiencing homelessness or housing insecurity and possible intervention functions to address barriers

**COM-B model component**[20]	**Theoretical domains constructs** [21]	**What needs to happen for the target behavior to occur?**	**Potential candidate intervention functions** [20]
**Physical capability**	Skills	Physical skills to take PrEP	Not applicable
**Psychological capability**	Knowledge	Need to know about PrEP and how to get/take it	Education about PrEP
	Cognitive & interpersonal skills	Need to be able to understand what is required to use PrEP, skills around navigating disclosure as needed	Education about PrEP, training in PrEP use and PrEP disclosure
	Memory, attention & decision processes	Need to remember to take PrEP and return for appointments	Training on setting reminder cues
**Physical opportunity**	Environmental context & resources	Need to be able to reach clinic (e.g., transport, money for bus), store PrEP or have a trusted person to watch belongings if living on the street, access to a phone to facilitate navigating the health system and receiving appointment reminders, complete laboratory procedures, and have other physical needs met (e.g., safety, food, trauma, mental health services, substance use services)	Environmental restructuring to alter physical settings of PrEP delivery, enablement to reduce physical barriers
**Social opportunity**	Social influences	Need to feel supported socially and reduce HIV- and PrEP-related stigma	Modeling of PrEP use and HIV prevention behaviors from others, enablement from health care personnel and other care team members
**Reflective motivation**	Beliefs about consequences	Need to perceive risk of HIV, trust the people providing PrEP and have rapport with health care providers, feel respected and supported by the PrEP providers, feel safe attending appointment	Motivational interviewing to take PrEP (e.g., persuasion)
	Optimism	Need to believe that PrEP will prevent HIV and lead to a healthy life	Education about PrEP
**Automatic motivation**	Reinforcement	Need to reinforce positive health behaviors (PrEP dosing, clinic attendance, laboratory procedures), cultural normativity around PrEP use	Incentives around PrEP use, clinic visit attendance, and other health indicators; persuasive messaging around PrEP

Surrounding the central hub of the BCW are nine “intervention functions” most likely to bring about individual-level behavior change for each determinant: education; restriction; coercion; persuasion; modeling; training; incentivization; environmental restructuring; and enablement [20]. We matched the appropriate intervention function to each determinant (column #4 in Table 1), and these functions offer a starting point for identifying broad categories of core components necessary for an implementation strategy to improve PrEP use among people experiencing homelessness and unstable housing.

### Review Methods

One author (JV) searched PubMed for primary studies with search terms related to HIV, PrEP, unstable housing or homelessness, and service delivery in either the title, abstract, and/or article keywords. Because the goal of this manuscript is to report on recent developments in the HIV prevention field, we restricted our search to studies published from June 1, 2014 – June 1, 2024. All co-authors reviewed case studies included and provided suggestions of other relevant citations that may have been missed in the initial search. We also searched abstract databases, restricted to the last five years, from the following international HIV conferences: Conference on Retroviruses and Opportunistic Infections (CROI), HIV Research for Prevention (HIVR4P), and the International AIDS Society Conference (IAS). JV reviewed all manuscripts and abstracts to assess relevancy for inclusion and abstracted data on the program, its target population and setting, the study design, outcomes assessed, and key findings. Where authors described program elements or evaluation outcomes related to the COM-B and BCW, this was also included in our extraction table. All articles that evaluated a PrEP implementation strategy for people experiencing homelessness or unstable housing were included as case studies (those that descriptively discussed barriers to PrEP use in this population, other literature reviews, or formative or intervention development research were not included).

## Review of the Literature

### Case Studies: ART and PrEP Implementation Approaches for People Living with Homelessness and Housing Insecurity

In this section, we ground our theoretical framing of determinants and intervention functions to address PrEP use with recent case studies of oral and long-acting ART and PrEP implementation approaches for people living with homelessness and housing insecurity in the United States. These case studies offer a data-driven approach to identify common components and their effects on HIV outcomes (Table 2). Findings presented here reflect published data but most of these programs are ongoing and evolving, and their findings and package of services may differ at the time of this manuscript publication.

**Table 2:** HIV treatment and prevention programs offering services to people experiencing homelessness or housing insecurity

Program	Target Population	Patient-Facing Package Components	Provider-Facing Package Components	Key Findings
POP-UP for oral ART delivery [12, 33]	Patients at Ward 86 in San Francisco who were experiencing homelessness or unstable housing; were off ART or viral load ≥ 200 copies/mL; missed ≥ 1 primary care visit in last year; and had ≥ 2 unscheduled Ward 86 visits in past year	***Low-threshold comprehensive primary care***: drop-in visit hours with no appointment needed; same-day ART restart, substance use treatment, primary care, and laboratory services; psychiatrist for phone consultations; optional medication pick-up directly from clinic ***Integrated social work and case management*:** Social services including care coordination, referrals for case management, and referrals for emergency and permanent housing assistance ***Incentives*:** $10 grocery store gift cards for weekly visits with provider or social worker or for laboratory draws; $25 grocery store gift cards for achieving or maintaining HIV viral load < 200 copies/mL every 3 months ***Enhanced outreach & care coordination:*** Patient navigator to find and link patients to care including benefits services, Medicaid enrolment, and medical appointments; POP-UP phone for text messages	In-service clinical trainings (e.g., on substance use treatment) Weekly case conferences (also attended by a psychiatrist) to facilitate care coordination and discussion Data dashboard for nurses to track primary care services, follow-up with patients, and coordinate with extended care team Quarterly team support sessions facilitated by a psychologist	• Enrolled 112 patients, of whom 52% lived outdoors, 100% had a substance use disorder, and 70% had a mental health disorder at enrollment • 70% had ≥ 1 POP-UP follow-up visit in each 4-month period • 59% had at least 1 gap between visits of ≥ 90 days • Median clinic visits in 12 months was 12 per patient (compared to median of 1 primary care visit and 3 drop-in visits at Ward 86 prior to enrollment) • Cumulative incidence of HIV viral suppression was 66% (95% CI: 57–74%) over 12 months • Baseline mental health diagnosis associated with lower risk of a ≥ 90 day gap between visits (53 versus 74%, OR: 0.40; 95% CI: 0.17–0.96)
MAX Clinic for oral ART delivery [13, 34]	Patients in Seattle who were off ART or viral load ≥ 200 copies/mL; poorly engaged in HIV care (no show visits in past year); and failed to re-engage in care after outreach attempts	***Low-threshold comprehensive primary care***: drop-in visit hours with no appointment needed; same-day ART restart, substance use treatment, Hepatitis C therapy; primary care, and laboratory services ***Integrated case management*:** Text message and direct phone access to case managers; case managers for care coordination and navigation ***Incentives*:** $10 food vouchers weekly; snacks at visits; no-cost bus passes; cell phones if needed; $25–50 once every two months for visits with blood draws; $50–100 for achieving or maintaining HIV viral load < 200 copies/mL every 2 months and a 1-time $100 bonus for a third consecutive suppressed viral load ***Enhanced outreach & care coordination:*** Case manager conducts outreach and serves as primary contacts for patients and providers and coordinated with release planning team at King County jails, housing, trauma and mental health case management agencies, medication adherence support programs, and office-based opioid treatment nurse managers and methadone providers	Low case load (~ 50 patients) per medical case manager Automated staff alerts when patients are seen in the emergency room or are admitted to a University of Washington Medicine system hospital Coordination between Max clinic staff and inpatient medical teams to plan transition to outpatient care	• Enrolled 50 patients, of whom 12% had unstable housing, 14% were sleeping in a shelter, and 38% were sleeping outside; 94% reported some substance use; and 78% had a psychiatric illness at enrollment • Among first 50 patients enrolled and 100 randomly selected Madison Clinic controls, viral suppression improved in both groups pre versus 1-year post (20 to 82% in Max patients, *p* < 0.001; 51% to 65% in controls, *p* = 0.04) • A larger improvement was seen in Max patients (aRRR = 3.2; 95% CI: 1.8–5.9) • Continuous viral suppression and care engagement improved in both cases and controls and did not differ between groups
HHOME for oral ART delivery [35]	People living with HIV in San Francisco with low CD4 count and detectable viral load, high emergency department and hospital utilization, no primary care, active substance use disorders, severe mental illness, and homelessness or unstable housing, based on HHOME’s Acuity & Chronicity Assessment	***Low-threshold comprehensive primary care***: care provided via mobile dyads at shelters, streets, encampments, hospitals, and treatment programs; staff also located at two drop-in clinics; HIV medications started as soon as possible along with other medications (psychiatric and addition medicine); weekly or directly observed dispensing by registered nurses or at methadone treatment programs ***Integrated case management*:** Clients placed in emergency stabilization at shelters, hotels, medical or psychiatric programs, or substance use treatment programs; permanent housing plans initiated immediately ***Enhanced outreach & care coordination:*** Recruitment via local hospitals, clinics, jails, law enforcement, HIV linkage, and community programs; interdisciplinary team includes medical provider, registered nurse, medical social worker, housing case manager, peer navigator, and program manager; persistent outreach and community engagement for lost or disengaged clients; bracelets with HHOME contact information for hospitals to notify the program; treatment starts in mobile care and then shifts to drop-in and clinic escorts; clients transitioned to less intensive case management after two in-clinic appointments	Weekly case conferences to facilitate care coordination and discussion Extensive team communication: morning phone check-ins; daily email summary; team text messages for urgent communication and integrated panel management	• From 2014–2017, enrolled 106 clients • At 12 months post-enrollment, 83.6% had two HIV primary care provider appointments, 60% were virally suppressed • 79% achieved viral suppression at some point in a one year period • 83.6% transitioned to stable placement and 73.8% entered supportive housing
AH3 for HIV testing and oral ART and PrEP linkage to care [36]	People experiencing homelessness and participating in daytime activities or overnight shelter at a homeless shelter in Jefferson County, Alabama	***Integrated case management*:** Placed a full-time HIV case manager at the shelter from a local AIDS service organization, who administered COVID-19 testing, HIV counseling and testing, and linkage to HIV care for individuals who tested positive for HIV. The case manager provided follow-up up until the day of the appointment and could provide transportation to the appointment as needed	Not specified	• From January 2021 – December 2021, 100% of shelter guests were offered an HIV test and only 2.7% declined testing • Identified 9 previously diagnosed and out of care people living with HIV and 1 newly diagnosed person • Four attended their primary care appointment within 30 days • 100% of guests who tested negative were counseled on PrEP and offered a PrEP referral but only 10 (1.4%) expressed interest and 1 linked to PrEP
SFDPH Whole Person Integrated Care long-acting ART and PrEP delivery [14, 45]	People experiencing homelessness or unstable housing engaged in low-barrier clinic- and community-based programs in San Francisco, CA	***Low-threshold, accessible LA-PrEP and ART integrated with primary care services***: Drop-in clinic hours 6-days per week without appointments; “direct-to-inject” approach without requiring oral medication lead in or baseline viral suppression for people living with HIV; on-site laboratory testing offered 6-days per week; mobile street-based outreach to administer injections off-site and provide reminders; extensive collaboration with Shelter Health staff and partnering case management programs ***Incentives*:** $10 gift cards for on-time injections and laboratory draws ***Enhanced outreach & care coordination:*** Follow-up and tracking by multidisciplinary team including physician, street-based outreach nurse, health workers, clinic nurse	In-service education on LA-PrEP and LA-ART for providers and staff and distribution of detailed protocols Weekly case conferences to facilitate discussion of individualized care plans, review upcoming injection due dates, and coordinate outreach	• From November 2021 to June 2024, initiated 41 patients on LA antiretrovirals (22 on LA ART and 19 on LA PrEP) • 27% were street homeless; 43% were sheltered homeless; 29% were unstably housed • Among 22 on LA ART, 15 initiated with detectable viremia and 9 had never been previously virally suppressed – all but 1 achieved or maintained virologic suppression (mean 14.4 months) • Among 19 on LA PrEP, all remained HIV negative (mean 8.9 months) • 8% LA-ART and 12% LA-PrEP injections were delayed > 1 week • LA PrEP was discontinued by 4 participants for risk change, loss to follow up, or care transfer
Boston Health Care for the Homeless Program (BHCHP) for oral PrEP delivery [15, 16]	People with sexual (e.g., transactional sex, condomless sex) or who use drugs (e.g., syringe sharing) that increase HIV risk among people experiencing homelessness in Boston, MA	***Community-driven PrEP education:*** Tailored education materials about PrEP; information from program PrEP navigator; print and word of mouth PrEP information dissemination to collaborating syringe service programs, homeless shelters, tent encampments, drug detoxification centers, and methadone clinics ***Low-threshold, accessible PrEP integrated with primary care services***: PrEP referral systems integrated with primary care, wound care, case management, behavioral health, treatment for opioid use disorder, and medical monitoring for over-sedation; Panel of “PrEP champions” (clinicians) and a PrEP nurse provide brief in-person or phone visits to review assessments and offer same-day PrEP prescribing; obtaining laboratory data through outreach-based phlebotomy; making referrals to other services as needed and desired; no PrEP discontinuations for missed visits as long as lab follow-up is performed ***Tailored prescribing supports:*** On-site pharmacies, short-term prescriptions (7–14 days), on-site medication storage at BHCHP and affiliated venues; assistance with medication pickup and delivery; nurse-facilitated daily medication dosing in outreach settings ***Intensive outreach & navigation:*** Full-time PrEP navigator; client follow-up via phone- and street-based outreach; accompanying clients to appointments; tailored navigation that could include weekly check-ins and lab follow-ups at four weeks and quarterly thereafter ***Trusting, respectful patient-provider relationships***:	Not specified	• From October 2018 to February 2020, enrolled 239 clients were linked to PrEP services and 152 were prescribed PrEP • This represented twice the number of prescriptions than the prior year • Cumulative probability of remaining on PrEP for 6 months was 44% (95% CI: 36%−52%) • Highly acceptable package
Ward 86 for long-acting PrEP delivery [37]	People attending Ward 86 clinic in San Francisco, who generally have high rates of homelessness, mental health issues, and substance use	***Low-threshold, accessible LA-PrEP with primary care services***: Drop-in visit hours with no appointment needed; “direct-to-inject” approach without requiring oral medication lead in; drop-in injection visits	Not specified	• From March 2022 – June 2023, initiated 30 patients on LA PrEP • 20% were homeless and 23% were unstably housed • 184 injections administered with median of 6 injections/person • 86% of injections were on time, 4% early, 8% late, and 2% late/not yet given • LA PrEP was discontinued by 3 participants for risk change, loss to follow-up, or care transfer • All remained HIV negative (median follow-up of 271 days)

*ART* antiretroviral therapy, *PrEP* pre-exposure prophylaxis, *LA* long-acting, *OR* odds ratio, 95%, *CI* 95% Confidence Interval, *aRRR* adjusted relative risk ratio

#### Oral ART Delivery for People Living with HIV

Three programs, POP-UP [12, 33], the MAX Clinic [13, 34], and HHOME [35] focus on low-barrier, high-touch care delivery to promote ART uptake and adherence among people living with HIV who are unstably housed. The POP-UP program is housed within Ward 86, the safety-net HIV clinic at San Francisco General Hospital, and operates through joint efforts from the San Francisco Department of Public Health (SFDPH) and the University of California. It provides low-barrier HIV primary care for unstably housed people who are viremic and have difficulty engaging in scheduled appointments [12, 33]. The program includes four patient-facing ART implementation approaches: low-barrier comprehensive care with integrated substance use and mental health care services; social work and case management including referrals for housing assistance; financial incentives; and enhanced outreach and care coordination [12, 33]. In addition, POP-UP includes provider-facing implementation approaches to facilitate patient tracking and care coordination including clinical trainings, weekly case conferences, data dashboards, and quarterly team support sessions [12, 33]. The program has enrolled a high-need cohort (e.g., 70% of patients had a substance use or mental health disorder) and reported improvements in clinic visit attendance and HIV viral suppression over a 12-month period compared with baseline [12, 33]. The MAX Clinic is housed within the Harborview Medical Center and is operated by the Madison Clinic and Public Health – Seattle & King County. It enrolls a similar cohort of patients with high viral loads and challenges attending clinic visits in Seattle, WA and also includes the same four patient-facing implementation components as in POP-UP along with three provider-facing components (low case loads with the assistance of a cadre of medical case managers, automated staff alerts for patient hospital admissions, and coordination to plan care transitions) [13, 34]. In a matched pairs analysis, enrollment in the MAX Clinic significantly improved viral suppression over a one-year period [13, 34]. The HIV Homeless-Health Outreach Mobile Engagement (HHOME) program is operated by the San Francisco Department of Public Health (SFDPH). It offers mobile care, with a mix of street- and clinic-based service delivery, to people who are experiencing homelessness or unstable housing and have detectable HIV viral loads, active substance use disorders or severe mental illness, and high emergency department or hospital utilization in San Francisco [35]. HHOME also includes mobile outreach-based primary care (with care in shelters, streets, encampments, hospitals, and treatment programs as well as at an HIV drop-in clinic), integrated substance use treatment and housing case management, but does not provide financial incentives [35]. Providers also attend weekly case conferences and engage in intensive team communication to coordinate patient care [35]. From 2014–2017, the majority of HHOME clients (79%) achieved HIV viral suppression in a one year period and 84% transitioned to stable housing. [35]

#### Status-Neutral ART and PrEP Delivery

Two programs, University of Alabama at Birmingham’s “Ending the HIV Epidemic: Addressing HIV Health and Homelessness” (AH3) [36] and the SFDPH’s Whole Person Integrated Care (WPIC) models [14], provide status-neutral HIV testing and linkage to oral and long-acting HIV treatment and prevention services (Table 2). Of the case studies described here, AH3 is unique in offering HIV services within homeless shelters by providing integrated case management, HIV counseling, and testing to people participating in daytime activities or staying overnight at a shelter in Alabama [36]. The program does not describe other patient- or provider-facing implementation approaches; however it does describe general operations. Over a one-year period, recent reports show that all shelter guests accepted HIV testing and were counseled on PrEP however only 40% of those who tested positive for HIV attended a clinic appointment within one month and only 1.4% of those who tested negative for HIV expressed interest in PrEP. [36]

The SFDPH WPIC program provides low-barrier oral and long-acting injectable ART and PrEP for people experiencing homelessness or unstable housing in San Francisco [14]. Patient-facing program components include “direct-to-inject” long-acting ART and PrEP (without the requirement for an oral medication lead in) integrated within primary care through a low-barrier (e.g., no appointments) central open-access clinic, satellite shelter health clinics, mobile street-based outreach, and partnerships with local community-based programs providing housing assistance [14]. The program also offers incentives for on-time injections and laboratory draws and enhanced monitoring and care coordination via multidisciplinary clinic- and street-based care providers [14]. Between 2021–2024, 22 clients initiated on long-acting ART (15 with detectable viremia) and all but 1 achieved and maintained viral suppression. In addition, 19 clients initiated long-acting PrEP, all of whom remained HIV negative [14]. Fewer than 10% of long-acting ART and PrEP injections were delayed overall.

#### Oral and Long-Acting PrEP Delivery

Two programs, Boston Health Care for the Homeless Program (BHCHP) [15, 16] and the Ward 86 clinic [37], developed programs focused on providing low-barrier PrEP to people experiencing homelessness or unstable housing (Table 2). The BHCHP program focuses on oral PrEP delivery for PEH engaging in risky sexual behavior or drug use and provides PrEP education and low-barrier PrEP integrated with primary care services, on-site medication storage, short-term refills, and intensive outreach and navigation support [15, 16]. It also explicitly emphasizes developing a culture of trusting and respectful patient-provider relationships to promote PrEP uptake and adherence [15, 16]. From 2018–2020, the program provided twice the number of PrEP prescriptions than in 2017 and, of 152 clients who received PrEP prescriptions, the cumulative probability of PrEP persistence through 6 months was 44%. [15, 16]

The Ward 86 program offers oral and long-acting PrEP to a patient population that includes those experiencing homelessness, mental health challenges, and substance use disorders via a “direct-to-inject” approach and use of drop-in appointments coupled with incentives and in-clinic provision of harm reduction supplies, along with a weekly multi-disciplinary panel management meeting [37]. It includes but is not limited to patients from the POPUP program, which is described above. As recently described, the program initiated 30 patients on long-acting PrEP in a 15-month period (from March 2022 to June 2023) and, similar to findings from the SF WPIC program, the majority of injections were on time and all patients remained HIV negative through follow-up. [37]

### The SHELTER Multicomponent Implementation Strategy for PrEP Provision among People Experiencing Homelessness and Unstable Housing

**Fig. 1:**
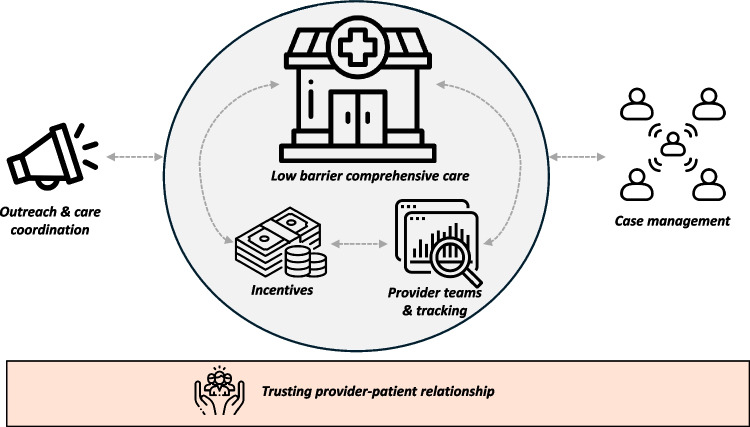
The “SHELTER” multi-component implementation strategy to promote oral and long-acting PrEP delivery among people experiencing homelessness or housing insecurity

Based on the behavior change targets theorized to promote PrEP use among people experiencing homelessness and unstable housing and case studies of ART and PrEP delivery models for this population, we propose the “SHELTER” (Supporting HIV prevention through Enhanced, Low-barrier, high-Touch services and Empowering Resources) multicomponent implementation strategy (Fig. 1). SHELTER comprises a minimum set of six core components to advance oral and long-acting PrEP delivery, identified by combining components found in most or all of the case studies that also meet criteria for candidate intervention functions identified in our BCW framework (Table 1). SHELTER components are applicable to all PrEP options: daily oral PrEP, event-driven oral PrEP, and injectable and long-acting PrEP options (which may be uniquely beneficial for unstably housed people given adherence challenges in this population).

In Table 3, we specify the individual elements of the SHELTER implementation strategy using a unified framework and established guidelines from the implementation science field for naming, defining, and operationalizing each to ensure that they are well understood and enable reproducibility [38]. Individual components of the SHELTER strategy have been derived from those described across case studies. Outreach, care coordination and street-based care coupled with low-barrier clinic spaces, are critical to reach individuals experiencing homelessness or housing insecurity and provide care. While traditional clinic-based care is generally more thorough, has more capacity, and can more effectively link patients to ancillary care, newer models of care delivery show that street-based care is an additional option particularly well-suited for people experiencing housing instability. Low-barrier comprehensive care and financial incentives for visits, laboratory testing, and PrEP adherence are critical to meet patients’ needs and promote continued engagement, while multidisciplinary provider meetings and data tracking are necessary to facilitate care coordination. Case management (with case managers based in clinics and in partner organizations in communities) can further assist patients in reaching PrEP goals and in linking to additional services to support with housing, substance use and mental health treatment, and trauma recovery. Underlying these components is the cultivation of a trusting relationship between the healthcare team and PrEP clients. By branding these components together into the SHELTER strategy, we hope to promote broader implementation of a comprehensive low-barrier, high-touch PrEP delivery approach that can be evaluated as a package and facilitate comparison of findings on PrEP outcomes across different settings and component operationalization approaches. We also provide suggestions of key implementation, service, and/or client-level outcomes that could be measured for each SHELTER strategy component, based on what has been measured in case studies including these components and the Proctor Implementation Outcomes Framework (“IOF”, which theorizes that implementation outcomes could lead to service outcomes which could in turn lead to client-level outcomes) [39]. These are meant to be starting points for evaluation planning evaluations of SHELTER components but we do not offer descriptions of how to operationalize each IOF outcome for each component.

**Table 3:** Specifying components [38] of the SHELTER implementation strategy to promote oral and long-acting PrEP delivery for people experiencing homelessness or housing insecurity

Strategy component name	Low-barrier comprehensive primary care	Integrated case management	Incentives	Enhanced outreach & care coordination	Provider team communication	Data tracking
**DEFINITION**	Any flexible PrEP delivery alongside other primary care services	Any care coordination for PrEP, other health care, and/or non-health care related services	Any monetary reward contingent on past behavior related to PrEP or health care engagement	Any navigation services to reach people outside of the clinic settings	Any in-person and/or virtual discussions to coordinate patient management	Any dashboards or alerts to monitor patient PrEP use and health outcomes
**ACTOR**	Clinician, nurse, pharmacist	Social worker, case manager	Clinician, nurse, social worker, case manager	Clinician, nurse, social worker, case manager, patient navigator, street-based outreach team	Clinician, nurse, pharmacist, psychologist, social worker, case manager	Clinician, nurse, pharmacist, psychologist, social worker, case manager
**ACTION**	Provide PrEP via a combination of drop-in visits with same day starts; a direct-to-inject approach for those interested in LA PrEP; telehealth visits as needed; onsite laboratory testing; assistance with PrEP pickup and medication storage; optional short-term prescriptions; and linkage to primary, trauma, mental health, and substance use care	Provide referrals for and warm handoffs to housing assistance, mental health care, trauma recovery, and substance use services; accompany clients to appointments as needed	Provide gift cards based on clinic appointment attendance or PrEP refills or on-time injections	Provide PrEP education in community settings (e.g., homeless shelters, tent encampments, syringe service programs); follow-up with PrEP clients regularly via phone- and street-based outreach and telehealth visits	Conduct emails, calls, and case conferences to discuss individualized care plans	Track patient follow-up visit attendance and receipt of other healthcare and housing/mental health/trauma/ substance use services
**ACTION TARGET** [40]	***PrEP client:*** environmental barriers to PrEP initiation and refills; resources ***Clinical team:*** organizational culture and climate	***PrEP client:*** resources; action planning; self-efficacy to access resources ***Clinical team:*** organizational culture and climate; professional boundaries and role	***PrEP client:*** reward behavior; client empowerment to engage	***PrEP client:*** social norms and pressure around PrEP; PrEP knowledge; environmental barriers to PrEP initiation and refills	***Clinical team:*** knowledge about clients’ needs; skills to engage with client; goals and action planning	***Clinical team:*** memory and decision-making around clients’ needs; monitoring
**TEMPORALITY**	As needed, throughout PrEP delivery	As needed, throughout PrEP delivery	Once every 2–3 months	As needed, throughout PrEP delivery	Daily as needed; once per week structured meetings	Daily as needed; once per week structured meetings
**DOSE**	Not applicable	Not applicable	$10 for appointment attendance, $25-$100 for refills, on-time injections, or adherence	Not applicable	15 min – 2 h per week, depending on team size and case load	15 min – 2 h per week, depending on team size and case load
**OUTCOMES** [39]	***Implementation outcomes:*** acceptability of PrEP for clients; appropriateness of PrEP and other healthcare services for clients; acceptability, feasibility, and sustainability of PrEP delivery for clinical team ***Service outcomes:*** effectiveness increasing uptake of and adherence to PrEP ***Client outcomes:*** Satisfaction with model; clinical symptoms	***Implementation outcomes:*** acceptability of other services for clients; penetration of other services for clients; fidelity to referral protocols by clinical team ***Client outcomes:*** Satisfaction with model	***Implementation outcomes:*** acceptability of PrEP for clients ***Service outcomes:*** effectiveness increasing adherence to PrEP	***Implementation outcomes:*** acceptability of PrEP for clients; penetration of PrEP in community for clients ***Service outcomes:*** effectiveness increasing uptake of and adherence to PrEP	***Implementation outcomes:*** fidelity to PrEP protocol; acceptability, feasibility, and sustainability of PrEP delivery for clinical team	***Implementation outcomes:*** fidelity to PrEP protocol; acceptability, feasibility, and sustainability of PrEP delivery for clinical team
**JUSTIFICATION**	Reduce and remove barriers to returning to the clinic, waiting at the clinic, and storing medications	Offer wrap-around services to address hierarchy of needs and provide holistic patient care	Provide reinforcement of positive behaviors	Meet PrEP clients where they are both physically and cognitively	Improve clinical skills and transfer of knowledge to enhance patient outcomes	Provide reinforcement of clinical progress and identify gaps

*PrEP* pre-exposure prophylaxis, *LA* long-acting

Low-barrier comprehensive care includes any model of flexible oral or long-acting PrEP delivery alongside other services to address barriers to PrEP in this population (e.g., harm reduction or ambulatory care services, drop-in clinic hours, telehealth visits, same-day starts, direct-to-inject long-acting PrEP, onsite options for laboratory testing and medication storage, and linkage to trauma recovery, mental health care and/or substance use treatment). However, it does not necessarily include full scope primary care which would be difficult and cost- and logistics-prohibitive to offer in many settings. Based on theoretical underpinnings of the BCW framework,[40] we hypothesize that the action target for this component is altering or reducing social and environmental barriers to PrEP, providing tangible resources for clients, and altering organizational culture and climate at the clinic level. Both status-neutral clinics (e.g., WPIC) and HIV-specific clinics (Ward 86) have PrEP programs, though primary care services are more comprehensively offered in the former. Moving forward, it would be ideal to equip both types of clinics for PrEP delivery using this comprehensive low-barrier care approach to address co-occurring housing, mental health, and substance use needs. Evaluating success of this component could include measuring changes in implementation outcomes like PrEP acceptability, and appropriateness of PrEP and other health care service delivery by clients and service outcomes like PrEP uptake and adherence. Secondary measures of success could include reductions in risky behavior, reductions in substance use, improvements in mental illness, and reductions in medical mistrust. Among providers, key implementation outcomes could include acceptability, feasibility, and sustainability of the model.

Integrated case management is defined as any care coordination for PrEP and/or other health or non-health (e.g., housing assistance) related services, which could include referrals and warm hand-offs for trauma recovery, mental health or substance use care. This component is hypothesized to target client resources, action planning around PrEP use and broader health and wellbeing goals, and self-efficacy to access resources. At the clinic level, it also could change organizational culture and climate around integrated service delivery and professional roles. Key implementation outcomes that might be affected by this component include acceptability of health care services broadly, penetration of PrEP (i.e. uptake of PrEP by those who could benefit from it), trauma, mental health, substance use, and housing assistance services, and fidelity to referral protocols.

Incentives include any reward contingent on health seeking behavior related to PrEP or care engagement. They are inconsistently included across our featured ART and PrEP delivery case studies, which may be due to limited resources. However, they are included here based on their effectiveness in PrEP and non-PrEP environments that include them, as well as their theoretical potential. Specifically, incentives reward client behavior; they theoretically motivate and empower PrEP clients to maintain engagement despite competing priorities related to other health risks and food, financial, and housing insecurity. The value of formal contingency management for PrEP adherence has been studied in multiple contexts,[41, 42] but PrEP programs will need to consider whether to and how best to structure an incentive program based on needs of clients who are experiencing housing insecurity.

Enhanced outreach and care coordination are defined as any navigation services to reach PrEP clients outside of clinic settings via, for example, street-based outreach teams or patient navigators. This component could include PrEP awareness and navigation in homeless shelters, tent encampments, and other community settings and provision of off-site injections or reminders regarding upcoming clinic-based injections to patients via phone, telehealth, or mobile street-based outreach. By increasing community knowledge about PrEP and improving communication pathways, this component could improve social norms around PrEP use. It also has the potential to reduce barriers to PrEP initiation and refills through access to PrEP navigators who can troubleshoot challenges outside of clinics. Potential implementation outcomes of this component include PrEP acceptability and PrEP penetration in communities of individuals who are homeless or unstably housed. Potential service outcomes include changes in PrEP uptake and adherence and durations of periods off PrEP.

The proposed SHELTER model includes two provider- or clinic-facing components: provider team communication and data tracking. Provider team communication includes any communication (e.g., in-person, phone-based, contact through the electronic health record) to coordinate patient management across multidisciplinary teams of health workers. Team communication strategies should attend to coordination needs within and between care delivery systems. Most case studies operationalize this as weekly case conferences to discuss individualized care plans, along with regular email and phone-based communication across providers. Regular and clear communication streams improve clinical knowledge about clients’ needs, skills to engage with the client across the provider team, and goals and action planning for the client’s PrEP use. Data tracking includes any data dashboards or automated alerts to monitor patient clinic visit attendance, PrEP use, or health outcomes. The MAX Clinic, POP-UP, and WPIC programs also use automated alerts (e.g., via Epic dashboards) to indicate when patients are seen in the emergency room or admitted to the hospital [13, 34], although these alerts may be difficult to implement at scale depending on their volume. Data dashboards and alerts could assist the provider team in decision-making around PrEP prescribing and other care and monitoring client’s care engagement. Key implementation outcomes of both the provider team communication and data tracking components include potential improvements in care engagement for services, improvements in care retention, fidelity to PrEP delivery protocols and increased acceptability, feasibility, and sustainability of PrEP delivery for people experiencing homelessness and unstable housing among clinical team members.

Underpinning all SHELTER components is the need to cultivate and maintain strong relationships of trust between PrEP clients and providers and between provider teams and community partners. For example, PrEP providers with BHCHP described the importance of trusting relationships with their clients in promoting safe spaces for conversations about PrEP clients’ broader needs and challenges [15]. While other case studies did not explicitly describe how they developed or maintained trusting relationships between provider teams and patients, it was clear that all created long-standing partnerships with community stakeholder groups and had deep ties with local organizations also providing substance use, trauma, mental health, and housing assistance to promote integrated service delivery. The importance of patient-provider relationships has also been found in quantitative research on preferences for HIV service delivery among PEH, who would trade financial gain for personal relationships with their care team [43]. By including trusting patient-provider relationships in our SHELTER model, we hope to encourage PrEP providers to recognize them as a necessary criteria for PrEP delivery among harder to reach populations and to specify ways that they can cultivate these relationships and measure their success over time in research and dissemination products.

### Future Directions

Our SHELTER multi-component implementation strategy offers a specified and comprehensive approach to oral and long-acting PrEP delivery programs for people experiencing homelessness and unstable housing but is based on a limited evidence base of case studies from the HIV prevention and treatment space. This review identifies a number of gaps and future research directions that are needed to maximize the utility and effectiveness of the SHELTER strategy on PrEP uptake and adherence in this population. The majority of case studies included here based their findings off pre-post test analyses and none disentangled the effectiveness or cost-effectiveness of specific ART and PrEP delivery components on uptake and adherence. There remains a need for consideration of adaptations and evaluations of the overall SHELTER strategy and its individual components, particularly given the time- and resource-intensiveness of this strategy. This review also has a number of limitations related to generalizability that need to be considered. First, this research literature and the included case studies primarily come from Democrat-led West Coast states with Medicaid expansion and funding for wrap-around health care services. While one case study was from a site in the southeastern United States, other information is not available on how components of SHELTER could be feasibly, acceptably, and effectively delivered in places with less funding and the highest HIV burden. Second, programs like Ryan White and Housing Opportunities for Persons with AIDS (HOPWA) provide funding for wrap-around healthcare services for people living with HIV but gaps remain in understanding how to offer status-neutral services for individuals eligible for and seeking HIV prevention medication alongside other housing and healthcare. Third, we were limited to reporting information from the published literature and conference abstracts, but there may be other programs offering PrEP in innovative, multi-component packages for people experiencing homelessness and unstable housing that are not published in peer-reviewed databases. Future work is needed to understand how wrap-around services are offered in different regions of the United States with different funding climates for HIV prevention, housing, mental health, and substance use service delivery to explore where SHELTER may be most applicable.

Evidence is mixed on the effectiveness of financial incentives on improving medication adherence, and it is unclear through which behavioral or cognitive pathways these incentives may work and what their ideal timing or amounts should be [18]. We include incentives in SHELTER but they may not be appropriate for all PrEP programs. Contingency management for PrEP adherence (e.g., using biomarkers of tenofovir adherence or other objective metrics) may also be empowering. Finally, all intervention functions identified using our COM-B framing are encompassed by one or more SHELTER components except for peer modeling around PrEP use. In populations with low PrEP awareness and high perceived or experienced PrEP stigma and medical mistrust, peers can be a welcoming and trusted source of PrEP information [30]. One recent pilot study conducted with US women offered PrEP education, counseling, and referral via peers at mobile syringe exchange sites and sex worker and syringe exchange drop-in centers [44]. Approximately 40% of this population reported housing insecurity at enrollment and, while PrEP interest was high (73%) after receipt of this peer intervention, only 6% attended an initial PrEP appointment and 0% received a PrEP prescription [44]. However, streamlined PrEP delivery approaches with peer navigators who could offer same-day PrEP services may mitigate some challenges with this peer approach.

We are at an exciting point of increased availability of injectable PrEP that can be administered once every two months in the United States, with other long-acting injectable and oral options on the horizon. These long-acting modalities can address many of the PrEP barriers related to capability (e.g., difficulty remembering to dose) and opportunity (e.g., lack of places to store medication) described in Table 1. However, long-acting PrEP is not a panacea to address intersectional determinants of health, including substance use, trauma, and mental health challenges. While the frequency of use and operationalization vary for oral versus long-acting PrEP, all SHELTER components apply for all current PrEP modalities with regular client contact and case management remaining necessary as long-acting PrEP is scaled up. Case studies also described the importance of same-day PrEP starts to reduce drop-offs between referrals, laboratory testing, and PrEP initiation. Long-acting PrEP programs offering same-day start have been found to be both safe and effective,[14, 37] although challenges exist with having clinic-based stock supply available and continued efforts to streamline long-acting PrEP initiation are critically needed among people experiencing homelessness and housing instability.

The SHELTER strategy focuses on implementation approaches to advance PrEP delivery in health care and community spaces by reducing barriers related to appointment constraints, wait times, and referrals for additional services among people who are experiencing homelessness and unstable housing and offering PrEP outreach, medication delivery and injections, laboratory draws, and patient navigator visits in trusted community locations. Several case studies (e.g., HHOME, WPIC, BHCHP) considered approaches to move PrEP into community-based locations like those also providing substance use interventions or homeless shelters. As one model of this, PrEP programs could leverage community partnerships to offer “prevention on demand”, where individuals signed up for PrEP through a clinic or mobile outreach could pick up medications at a local pharmacy or other community location of their choice. Two pilot studies offered PrEP information, counseling, and referrals at community locations, although they had very low PrEP uptake [36, 44] indicating that more work is needed to translate knowledge about PrEP to PrEP uptake among individuals experiencing homelessness and unstable housing. Co-locating PrEP providers, who can offer same-day pills and injections, at venues where these individuals already are (e.g., substance use treatment programs, syringe exchange programs, soup kitchens, homeless shelters), remains critical. As an example of this the WPIC program in San Francisco offers direct-to-inject long-acting PrEP in shelters, tents, and other community-based locations [45]. The outreach, care coordination, case management, and provider tracking and communication aspects of the SHELTER strategy can apply to these de-medicalized contexts to offer low-barrier comprehensive care with PrEP outside of health clinic settings.

## Conclusions

Gaps in PrEP knowledge, uptake, and adherence among people experiencing homelessness and unstable housing have contributed to shortfalls in meeting Ending the Epidemic goals in the United States. PrEP programs must address barriers related to PrEP capabilities, opportunities, and motivations, including challenges getting to a clinic and challenges remembering and storing PrEP and accessing health care services, to effectively reduce HIV incidence in this population. We synthesized evidence from recent case studies offering HIV treatment and prevention services to people experiencing homelessness and unstable housing to develop the SHELTER multi-component implementation model of low-barrier, high-touch oral and long-acting PrEP delivery. Our proposed SHELTER model is a starting point for future PrEP research on the effectiveness and cost-effectiveness of the overall approach, individual model components, and their generalizability to PrEP delivery outside of health care clinic spaces.

## Data Availability

No datasets were generated or analysed during the current study.

## References

[1] Berthaud V, Johnson L, Jennings R, et al. The effect of homelessness on viral suppression in an underserved metropolitan area of middle Tennessee: potential implications for ending the HIV epidemic. BMC Infect Dis. 2022;22(1):144. 10.1186/s12879-022-07105-y. 35144557 10.1186/s12879-022-07105-y PMC8830956

[2] de Sousa T, Andrichik A, Prestera E, et al. The 2023 Annual Homelessness Assessment Report (AHAR) to Congress. The US Department of Housing and Urban Development (HUD); 2023.

[3] Bauer GR. Incorporating intersectionality theory into population health research methodology: challenges and the potential to advance health equity. Soc Sci Med. 2014;110:10–7. 10.1016/j.socscimed.2014.03.022. 24704889 10.1016/j.socscimed.2014.03.022

[4] Bowleg L. The problem with the phrase women and minorities: intersectionality-an important theoretical framework for public health. Am J Public Health. 2012;102(7):1267–73. 10.2105/AJPH.2012.300750. 22594719 10.2105/AJPH.2012.300750 PMC3477987

[5] Kushel MB, Vittinghoff E, Haas JS. Factors associated with the health care utilization of homeless persons. JAMA. 2001;285(2):200–6. 10.1001/jama.285.2.200. 11176814 10.1001/jama.285.2.200

[6] Kushel MB, Gupta R, Gee L, Haas JS. Housing instability and food insecurity as barriers to health care among low-income Americans. J Gen Intern Med. 2006;21(1):71–7. 10.1111/j.1525-1497.2005.00278.x. 16423128 10.1111/j.1525-1497.2005.00278.x PMC1484604

[7] Shubber Z, Mills EJ, Nachega JB, et al. Patient-reported barriers to adherence to antiretroviral therapy: a systematic review and meta-analysis. PLoS Med. 2016;13(11):e1002183. 10.1371/journal.pmed.1002183. 27898679 10.1371/journal.pmed.1002183 PMC5127502

[8] Aidala AA, Wilson MG, Shubert V, et al. Housing status, medical care, and health outcomes among people living with HIV/AIDS: a systematic review. Am J Public Health. 2016;106(1):e1–23. 10.2105/AJPH.2015.302905. 26562123 10.2105/AJPH.2015.302905 PMC4695926

[9] McMahon JM, Braksmajer A, Zhang C, et al. Syndemic factors associated with adherence to antiretroviral therapy among HIV-positive adult heterosexual men. AIDS Res Ther. 2019;16(1):32. 10.1186/s12981-019-0248-9. 31706357 10.1186/s12981-019-0248-9 PMC6842154

[10] Clemenzi-Allen A, Geng E, Christopoulos K, et al. Degree of housing instability shows independent “dose-response” with virologic suppression rates among people living with Human Immunodeficiency Virus. Open Forum Infect Dis. 2018;5(3):ofy035. 10.1093/ofid/ofy035. 29577059 10.1093/ofid/ofy035 PMC5850870

[11] Henwood BF, Rhoades H, Redline B, Dzubur E, Wenzel S. Risk behaviour and access to HIV/AIDS prevention services among formerly homeless young adults living in housing programmes. AIDS Care. 2020;32(11):1457–61. 10.1080/09540121.2019.1699643. 31791132 10.1080/09540121.2019.1699643 PMC7263964

[12] Hickey MD, Imbert E, Appa A, et al. HIV treatment outcomes in POP-UP: drop-in HIV primary care model for people experiencing homelessness. J Infect Dis. 2022;226(Supplement_3):S353–62. 10.1093/infdis/jiac267. 35759251 10.1093/infdis/jiac267 PMC10233499

[13] Dombrowski JC, Ramchandani M, Dhanireddy S, Harrington RD, Moore A, Golden MR. The Max Clinic: medical care designed to engage the hardest-to-reach persons living with HIV in Seattle and King County Washington. AIDS Patient Care STDs. 2018;32(4):149–56. 10.1089/apc.2017.0313. 29630852 10.1089/apc.2017.0313 PMC5905858

[14] Mehtani NJ, Strough A, Strieff S, et al. Feasibility of implementing a low-barrier long-acting injectable antiretroviral program for HIV treatment and prevention for people experiencing homelessness. JAIDS. 2024;96(1):61–7. 10.1097/QAI.0000000000003396. 38346426 10.1097/QAI.0000000000003396 PMC11009050

[15] Bazzi AR, Shaw LC, Biello KB, Vahey S, Brody JK. Patient and provider perspectives on a novel, low-threshold HIV PrEP program for people who inject drugs experiencing homelessness. J Gen Intern Med. 2023;38(4):913–21. 10.1007/s11606-022-07672-5. 35614171 10.1007/s11606-022-07672-5 PMC9132566

[16] Biello KB, Bazzi AR, Vahey S, Harris M, Shaw L, Brody J. Delivering preexposure prophylaxis to people who use drugs and experience homelessness, Boston, MA, 2018–2020. Am J Public Health. 2021;111(6):1045–8. 10.2105/AJPH.2021.306208. 33950728 10.2105/AJPH.2021.306208 PMC8101577

[17] WHO. Differentiated and Simplified Pre-Exposure Prophylaxis for HIV Prevention: Update to WHO Implementation Guidance. World Health Organization; 2022.

[18] Clemenzi-Allen AA, Hickey M, Conte M, et al. Improving care outcomes for PLWH experiencing homelessness and unstable housing: a synthetic review of clinic-based strategies. Curr HIV/AIDS Rep. 2020;17(3):259–67. 10.1007/s11904-020-00488-0. 32382919 10.1007/s11904-020-00488-0 PMC8045147

[19] Ehrenkranz P, Grimsrud A, Rabkin M. Differentiated service delivery: navigating the path to scale. Curr Opin HIV AIDS. 2019;14(1):60–5. 10.1097/COH.0000000000000509. 30394947 10.1097/COH.0000000000000509

[20] Michie S, van Stralen MM, West R. The behaviour change wheel: a new method for characterising and designing behaviour change interventions. Implement Sci. 2011;6:42. 10.1186/1748-5908-6-42. 21513547 10.1186/1748-5908-6-42 PMC3096582

[21] Atkins L, Francis J, Islam R, et al. A guide to using the Theoretical Domains Framework of behaviour change to investigate implementation problems. Implement Sci. 2017;12(1):77. 10.1186/s13012-017-0605-9. 28637486 10.1186/s13012-017-0605-9 PMC5480145

[22] Barker F, Atkins L, de Lusignan S. Applying the COM-B behaviour model and behaviour change wheel to develop an intervention to improve hearing-aid use in adult auditory rehabilitation. Int J Audiol. 2016;55(sup3):S90–8. 10.3109/14992027.2015.1120894. 27420547 10.3109/14992027.2015.1120894

[23] Mangurian C, Niu GC, Schillinger D, Newcomer JW, Dilley J, Handley MA. Utilization of the behavior change wheel framework to develop a model to improve cardiometabolic screening for people with severe mental illness. Implement Sci. 2017;12(1):134. 10.1186/s13012-017-0663-z. 29137666 10.1186/s13012-017-0663-z PMC5686815

[24] Handley MA, Harleman E, Gonzalez-Mendez E, et al. Applying the COM-B model to creation of an IT-enabled health coaching and resource linkage program for low-income Latina moms with recent gestational diabetes: the STAR MAMA program. Implement Sci. 2016;11(1):73. 10.1186/s13012-016-0426-2. 27193580 10.1186/s13012-016-0426-2 PMC4870786

[25] Dahlby L, Boyd J, Knight R, et al. The perspectives of street-involved youth who use drugs regarding the acceptability and feasibility of HIV pre-exposure prophylaxis: a qualitative study. AIDS Care. 2023;35(4):480–7. 10.1080/09540121.2022.2085868. 35698454 10.1080/09540121.2022.2085868 PMC9930182

[26] Shaw LC, Biello KB, Vahey S, Brody JK, Bazzi AR. PrEP disclosure and discussions within social networks of people who inject drugs experiencing homelessness: a brief report. BMC Public Health. 2023;23(1):263. 10.1186/s12889-023-15153-5. 36750814 10.1186/s12889-023-15153-5 PMC9903274

[27] Gregg E, Linn C, Nace E, Gelberg L, Cowan B, Fulcher JA. Implementation of HIV preexposure prophylaxis in a homeless primary care setting at the veterans affairs. J Prim Care Community Health. 2020;11:2150132720908370. 10.1177/2150132720908370. 32100617 10.1177/2150132720908370 PMC7045292

[28] Bonett S, Mahajan A, da Silva DT, et al. Advancing the community plan to end the HIV Epidemic in Philadelphia: a qualitative descriptive evaluation of low-threshold PrEP services in sexual health clinics. Implement Sci Commun. 2024;5(1):4. 10.1186/s43058-023-00543-y. 38183133 10.1186/s43058-023-00543-y PMC10768374

[29] Andrzejewski J, Pines HA, Morris S, et al. Determinants of HIV pre-exposure prophylaxis (PrEP) retention among transgender women: a sequential, explanatory mixed methods study. Int J Environ Res Public Health. 2024;21(2):133. 10.3390/ijerph21020133. 38397624 10.3390/ijerph21020133 PMC10888369

[30] Mayer KH, Chan PA, R Patel R, Flash CA, Krakower DS. Evolving models and ongoing challenges for HIV preexposure prophylaxis implementation in the United States. JAIDS. 2018;77(2):119–127. 10.1097/QAI.0000000000001579. 10.1097/QAI.0000000000001579 PMC5762416 29084044

[31] Santa Maria D, Flash CA, Narendorf S, et al. Knowledge and attitudes about pre-exposure prophylaxis among young adults experiencing homelessness in seven U.S. cities. J Adolesc Health. 2019;64(5):574–80. 10.1016/j.jadohealth.2018.06.023. 30254009 10.1016/j.jadohealth.2018.06.023

[32] Storholm ED, Klein DJ, Pedersen ER, et al. Sociodemographic and behavioral risk correlates of PrEP interest and use among young adults experiencing homelessness in Los Angeles. AIDS Behav. 2024;28(4):1216–26. 10.1007/s10461-023-04144-7. 37698638 10.1007/s10461-023-04144-7 PMC10927607

[33] Imbert E, Hickey MD, Clemenzi-Allen A, et al. Evaluation of the POP-UP programme: a multicomponent model of care for people living with HIV with homelessness or unstable housing. AIDS. 2021;35(8):1241–6. 10.1097/QAD.0000000000002843. 34076613 10.1097/QAD.0000000000002843 PMC8186736

[34] Dombrowski JC, Galagan SR, Ramchandani M, et al. HIV care for patients with complex needs: a controlled evaluation of a walk-in, incentivized care model. Open Forum Infect Dis. 2019;6(7):ofz294. 10.1093/ofid/ofz294. 31341930 10.1093/ofid/ofz294 PMC6641789

[35] Borne D, Tryon J, Rajabiun S, Fox J, de Groot A, Gunhouse-Vigil K. Mobile multidisciplinary HIV medical care for hard-to-reach individuals experiencing homelessness in San Francisco. Am J Public Health. 2018;108(S7):S528–30. 10.2105/AJPH.2018.304732. 32941772 10.2105/AJPH.2018.304732 PMC6290579

[36] Kay ES, Bruce J, Foster-Hill S, Rygiel A, Batey DS. Addressing HIV and homelessness during COVID-19: a community-based demonstration project. Health Promot Pract. 2024;25(2):178–82. 10.1177/15248399221135589. 36448342 10.1177/15248399221135589 PMC9713533

[37] Bisom-Rapp E, Camp C, Oskarsson J, et al. Rapid long-acting injectable PrEP implementation in a vulnerable urban safety net clinic population. Poster Presentation presented at: CROI 2023; March 3, 2024; Denver, CO.

[38] Proctor EK, Powell BJ, McMillen JC. Implementation strategies: recommendations for specifying and reporting. Implement Sci. 2013;8(1):139. 10.1186/1748-5908-8-139. 24289295 10.1186/1748-5908-8-139 PMC3882890

[39] Proctor E, Silmere H, Raghavan R, et al. Outcomes for implementation research: conceptual distinctions, measurement challenges, and research agenda. Adm Policy Ment Health. 2011;38(2):65–76. 10.1007/s10488-010-0319-7. 20957426 10.1007/s10488-010-0319-7 PMC3068522

[40] Cane J, O’Connor D, Michie S. Validation of the theoretical domains framework for use in behaviour change and implementation research. Implement Sci. 2012;7(1):37. 10.1186/1748-5908-7-37. 22530986 10.1186/1748-5908-7-37 PMC3483008

[41] Sung ML, Viera A, Esserman D, et al. Contingency Management and Pre-Exposure Prophylaxis Adherence Support Services (CoMPASS): A hybrid type 1 effectiveness-implementation study to promote HIV risk reduction among people who inject drugs. Contemp Clin Trials. 2023;125:107037. 10.1016/j.cct.2022.107037. 36460267 10.1016/j.cct.2022.107037 PMC9918697

[42] Mitchell JT, Burns CM, Atkinson B, et al. Feasibility, Acceptability, and preliminary efficacy of a gamified mobile health contingency management intervention for PrEP adherence among Black MSM. AIDS Behav. 2022;26(10):3311–24. 10.1007/s10461-022-03675-9. 35416595 10.1007/s10461-022-03675-9 PMC9474612

[43] Conte M, Eshun-Wilson I, Geng E, et al. Brief report: understanding preferences for HIV care among patients experiencing homelessness or unstable housing: a discrete choice experiment. JAIDS. 2020;85(4):444–9. 10.1097/QAI.0000000000002476. 33136742 10.1097/QAI.0000000000002476 PMC8028840

[44] Blackstock OJ, Platt J, Golub SA, et al. A pilot study to evaluate a novel pre-exposure prophylaxis peer outreach and navigation intervention for women at high risk for HIV infection. AIDS Behav. 2021;25(5):1411–22. 10.1007/s10461-020-02979-y. 32748159 10.1007/s10461-020-02979-y PMC8284566

[45] Mehtani NJ, Strough A, Strieff S, Martinez C, Eveland J, Zevin B. Low-barrier long-acting injectable antiretrovirals for HIV treatment and prevention among people who use drugs. Oral Presentation at. CDPP; June 19, 2024; Montreal, Canada.

